# What are the causes for low birthweight in Japan? A single hospital-based study

**DOI:** 10.1371/journal.pone.0253719

**Published:** 2021-06-23

**Authors:** Yoshifumi Kasuga, Satoru Ikenoue, Masumi Tamagawa, Maki Oishi, Toyohide Endo, Yu Sato, Miho Iida, Yasunori Sato, Mamoru Tanaka, Daigo Ochiai

**Affiliations:** 1 Department of Obstetrics and Gynaecology, Keio University School of Medicine, Tokyo, Japan; 2 Department of Preventive Medicine and Public Health, Keio University School of Medicine, Tokyo, Japan; Kobe University Graduate School of Medicine School of Medicine, JAPAN

## Abstract

Low-birthweight (LBW; <2,500 g) babies are at a higher risk of poor educational achievement, disability, and metabolic diseases than normal-birthweight babies in the future. However, reliable data on factors that contribute to LBW have not been considered previously. Therefore, we aimed to examine the distribution of the causes for LBW. A retrospective review of cases involving 4,224 babies whose mothers underwent perinatal care at Keio University Hospital between 2013 and 2019 was conducted. The LBW incidence was 24% (1,028 babies). Of the 1,028 LBW babies, 231 babies were from multiple pregnancies. Of the 797 singleton LBW babies, 518 (65%) were born preterm. Obstetric complications in women with preterm LBW babies included premature rupture of membrane or labor onset (31%), hypertensive disorders of pregnancy (HDP, 64%), fetal growth restriction (24%), non-reassuring fetal status (14%), and placental previa/vasa previa (8%). Of the 279 term LBW babies, 109 (39%) were small for gestational age. Multiple logistic regression analyses revealed the following factors as LBW risk factors in term neonates: low pre-pregnancy maternal weight, inadequate gestational weight gain, birth at 37 gestational weeks, HDP, anemia during pregnancy, female sex, and neonatal congenital anomalies. HDP was an LBW risk factor not only in preterm births but also in term births. Our results suggest that both modifiable and non-modifiable factors are causes for LBW. It may be appropriate to consider a heterogeneous rather than a simple classification of LBW and to evaluate future health risks based on contributing factors.

## Introduction

Low birthweight (LBW) is defined as a birthweight < 2,500 g, and the proportion of neonates identified as having LBW during the past decade is reported to be approximately 10% in Japan. Neonates with LBW at delivery are known to have a higher risk of not only infant morbidity and mortality but also poor educational achievement, disability, and the development of metabolic diseases (i.e., type 2 diabetes, cardiovascular disease, and hypertension) in the future [1–4]. A recent report has indicated that LBW is associated with an increased risk of type 2 diabetes in Japanese adults [5]. Therefore, Normile D (2018) raised concerns regarding the future of Japanese healthcare in relation to the increasing number of neonates with LBW [6]. In the United Kingdom, 14 risk factors for LBW have been reported (i.e., drug use, smoking during pregnancy, low body mass index [BMI], teenage pregnancy, and several perinatal complications) [7]. Studies on East Asians have indicated that assisted reproductive technology is associated with LBW, though the mechanism is unclear [8,9]. Additionally, preterm birth is strongly associated with LBW, and small for gestational age (SGA: birthweight < the 10th percentile) is worthy of attention as a cause for LBW. In previous studies, inadequate gestational weight gain (GWG) and maternal underweight (pre-pregnancy BMI < 18.5 kg/m^2^) were found to be associated with small for gestational age (SGA: a birthweight of < the 10th percentile), including LBW, among Japanese pregnant women [10–14]. We were concerned regarding the fact that the reports describing the future risks of LBW did not mention several of the causes of LBW even though LBW can be observed in a mixed population of individuals.

There is no doubt that LBW should be prevented for effective lifelong healthcare in the future; however, there are still limited reliable data on causes for LBW in Japanese pregnancies. Moreover, the identification of modifiable factors responsible for LBW is important so that appropriate interventions could be administered during perinatal care. Therefore, in this study, we aimed to investigate the recent distribution of the causes for LBW in our hospital, which is located in the central area of Tokyo, Japan, to reduce the prevalence of LBW.

## Materials and methods

### Study population

Data associated with pregnancies in Japanese women who underwent perinatal care at the Keio University Hospital between January 2013 and December 2019 were retrospectively reviewed. The exclusion criteria were pregnancy loss before 22 weeks of gestation and intrauterine fetal death. Details regarding the maternal and neonatal characteristics and perinatal outcomes were collected from the medical records of our hospital. This research was performed in accordance with the Declaration of Helsinki, and the written informed consent was obtained from the patients. This study was approved by the Ethics committee of the Keio University School of Medicine, Tokyo, Japan (No. 20150103, approved June 23, 2015).

### Birth outcomes

Gestational age was confirmed during the first trimester using crown-rump length measurements. Perinatal management of pregnancy-related symptoms (i.e., premature labor, premature rupture of membranes [PROM], gestational diabetes, and hypertensive disorders of pregnancy [HDP]) was performed at the discretion of each obstetrician based on the clinical recommendations of the Japan Society of Obstetrics and Gynecology (JSOG) [15]. In our hospital, all mothers with multiple pregnancies deliver via cesarean section (CS). All patients with malpresentation or histories of prior CS or uterine surgery (i.e., myomectomy, adenomyomectomy, and radical trachelectomy [RT]) were planned for elective CS at 37–38 gestational weeks. Further, CS was performed at 34–37 gestational weeks in patients with asymptomatic placental previa or vasa previa. Fetal growth restriction (FGR) was defined as a mean (SD) estimated fetal weight of < -1.5 [15]. Using the Japanese standard sex- and parity-specific birthweight percentile curves, a birthweight of ≥ the 90th percentile was defined as large for gestational age and a birthweight of < the 10th percentile was designated as SGA [16]. HDP was defined as a systolic blood pressure of 140 mmHg or more or a diastolic blood pressure of 90 mmHg or more observed on at least two occasions occurring at least 4 hours apart in a patient who was normotensive prior to 20 gestational weeks [15]. Non-reassuring fetal status (NRFS) was defined as an abnormal fetal heart rate pattern on a non-stress test [15]. In the case of a singleton pregnancy, we calculated the expected GWG at 40 gestational weeks using a previously reported method that was validated in a Japanese population [11,14]. The expected appropriate maternal GWG was evaluated using the Institute of Medicine criteria for underweight (12.7 kg ≤ GWG ≤ 18.1 kg), normal weight (18.5 kg/m^2^ ≤ BMI < 25.0 kg/m^2^: 11.3 kg ≤ GWG ≤ 15.9 kg), overweight (25.0 kg/m^2^ ≤ BMI < 30.0 kg/m^2^: 6.8 kg ≤ GWG ≤ 11.3 kg), or obese (30.0 kg/m^2^ ≤ BMI: 5.0 kg ≤ GWG ≤ 9.1 kg) patients [17]. Anemia during pregnancy was defined as a hemoglobin (Hb) level < 10 mg/dL and was treated with medication.

### Statistical analysis

Data are presented as the median (range) or number of cases (percentage). Continuous data were compared between groups using the Mann–Whitney *U* test. Categorical variables were analyzed with the chi-square or Fisher’s exact test. The pregravid BMI and GWG trends were analyzed using the Cochran–Armitage trend analysis. In all tests, *P* < 0.05 was considered significant. A multiple logistic regression analysis was performed to evaluate the relative contributions of the various maternal and perinatal factors to LBW in neonates delivered at term. The control group consisted of all pregnant women who underwent perinatal care at our hospital between January 2013 and December 2019 and who delivered singleton babies with birthweights of over 2,500 g at term (n = 2,965). The following independent variables were included in the model based on the clinical relevance and statistical significance of their association with LBW: maternal age at delivery, method of conception, pre-pregnancy BMI, GWG, gestational week at delivery, mode of delivery, presence of HDP, anemia during pregnancy, neonatal congenital anomaly, and offspring sex. Odds ratios (OR) and their 95% confidence intervals (CI) were evaluated for the associations between LBW and the aforementioned clinical features. Statistical analyses were performed using the JMP software (ver. 15, SAS Inst. Inc., Cary, NC).

## Results

### Trends of LBW

**Fig 1:**
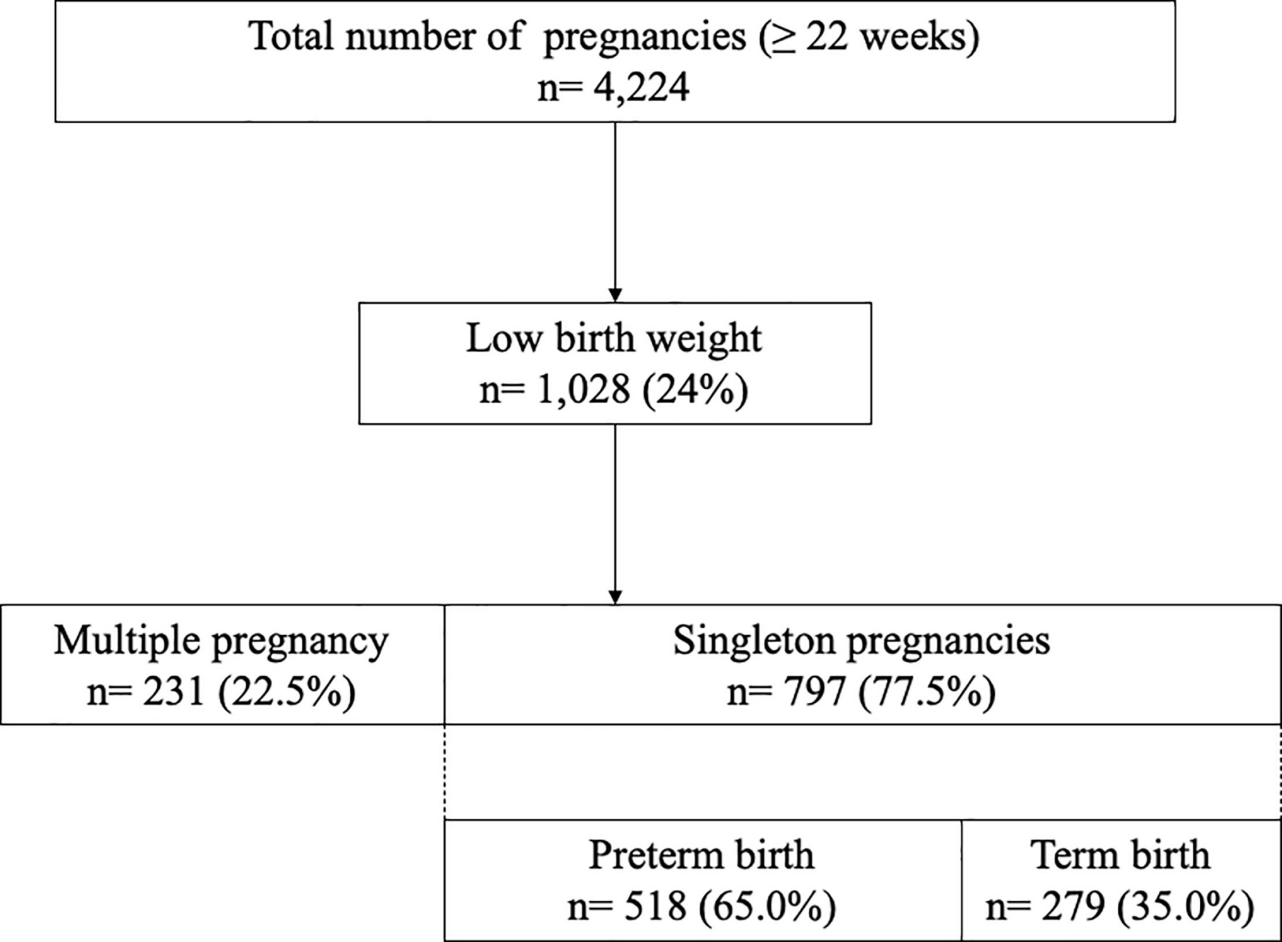
Flowchart for inclusion of pregnant women with low birthweight infants.

Of the 4,224 babies who were born during the study period, 1,028 (24%) were born with LBW. The flowchart for the inclusion of pregnant women who delivered babies with LBW is depicted in Fig 1. During the study period, there were 276 babies born from multiple pregnancies (two triplets and 135 twins) and 22.5% of the babies with LBW were from multiple pregnancies. Moreover, of the 276 babies from multiple pregnancies, 231(84%) were born with LBW. Of the 797 singleton babies born with LBW, 518 babies (65%) were born before 37 gestational weeks and 279 babies (35%) were born after 37 gestational weeks.

### Multiple pregnancies

The maternal and neonatal characteristics in the cases involving multiple pregnancies are presented in Tables 1 and 2. Of the two triplet pregnancies, one was delivered at 25 gestational weeks and the other at 32 gestational weeks, with all the babies born with LBW. Of the 225 LBW twin babies, there were no monochorionic-monoamniotic twin babies, 96 monochorionic-diamniotic twin babies, and 129 dichorionic-diamniotic twin babies. Of the 130 mothers with multiple pregnancies, 42 women conceived using *in vitro* fertilization-embryo transfer (IVF-ET). The median gestational weeks at delivery was 35 weeks (range: 22–38), and there were 200 preterm births. Eighty-five babies (37%) from multiple pregnancies were SGA.

**Table 1:** Maternal characteristics in multiple pregnancy.

Maternal Characteristics (n = 130)			
Monochorionic-monoamniotic twin		0	(0%)
Monochorionic-diamniotic twin		54	(41%)
Dichorionic-diamniotic twin		74	(57%)
Triplet pregnancy		2	(2%)
Maternal age at delivery	(years)	35	(23–55)
40 or older		20	(15%)
35 to 39		48	(37%)
Maternal pre-pregnancy BMI	(kg/m^2^)	20.2	(15.4–33.1)
Underweight (BMI< 18.5)		19	(15%)
Normal weight (18.5≤ BMI< 25.0)		97	(74%)
Overweight (25.0≤ BMI< 30.0)		10	(8%)
Obese (30≤ BMI)		1	(1%)
Unknown		3	(2%)
Nulliparity		91	(70%)
Smoking during pregnancy		0	(0%)
Method of conception			
Spontaneous conception		88	(68%)
IVF-ET		42	(32%)
Perinatal complications			
Twin-to-twin transfusion syndrome		2	(2%)
Gestational diabetes		26	(20%)
Preterm PROM		14	(11%)
Hypertensive disorder in pregnancy		13	(10%)
HELLP syndrome		2	(2%)
Placental abruption		1	(1%)
Placental previa		0	(0%)
Elective cesarean section		71	(55%)
Emergency cesarean section		59	(45%)

BMI: Body mass index, IVF-ET: *in-vitro* fertilization-embryo transfer, PROM: Premature rupture of membrane. Data were median (range) or n (%).

**Table 2:** Neonatal characteristics in multiple pregnancy.

Neonatal characteristics (n = 231)			
Gestational weeks at delivery	(weeks)	36	(22–37)
Preterm birth (< 37 gestational weeks)		202	(87%)
Offspring sex (female)		119	(52%)
Birthweight	(g)	2007	(461–2496)
Small for gestational age		85	(37%)
Large for gestational age		2	(1%)

Data were median (range) or n (%).

### Singleton babies with LBW delivered before 37 gestational weeks

The maternal characteristics of mothers who underwent preterm childbirth are shown in Table 3. In the case of singleton pregnancies, the obstetric complications of the women with preterm LBW babies included preterm PROM or labor onset (31%), HDP (64%), FGR (24%), NRFS (14%), and placental previa or vasa previa (8%). There were 16 mothers with a history of conization for cervical lesions (3%), and 28 pregnancies occurred after RT for early cervical cancer (5%). In the case of the three mothers with invasive cervical cancer, five with ovarian cancer, one with breast cancer, and one with a brain tumor, we performed elective CS during the late preterm period to start the administration of cancer treatments immediately. Elective CS was performed for five mothers during the late preterm period because of maternal complications (i.e., Crohn’s disease [n = 1], ileus [n = 1], postoperative biliary atresia [n = 1], giant uterine myoma [n = 1], deep vein thrombosis [n = 1], and epilepsy [n = 2]). Furthermore, of 40 mothers with fetal anomalies, CS was performed in 17 cases during the preterm period to administer neonatal treatment for fetal conditions (i.e., pleural effusion [n = 8], duodenal atresia [n = 3], hepatomegaly [n = 1], cloacal malformation [n = 1], gastroschisis [n = 3], and cardiac rhabdomyoma [n = 1]); however, causes for others were NRFS or maternal perinatal complication.

**Table 3:** Maternal and neonatal characteristics in single pregnancy at preterm delivery.

n = 518			
Maternal age at delivery	(years)	35	(16–53)
40 or older		71	(14%)
35 to 39		181	(35%)
Teenager		1	(0%)
Maternal pre-pregnancy BMI	(kg/m^2^)	20.3	(14.4–37.5)
Underweight (BMI< 18.5)		99	(19%)
Normal weight (18.5≤ BMI< 25.0)		355	(69%)
Overweight (25.0≤ BMI< 30.0)		31	(6%)
Obese (30≤ BMI)		8	(1%)
Unknown		25	(5%)
Nulliparity		346	(67%)
Method of conception			
Spontaneous conception		377	(73%)
IVF-ET		141	(27%)
Gestational weeks at delivery	(weeks)	34	(22–36)
Mode of delivery			
Vaginal delivery		102	(20%)
Cesarean section		416	(80%)
Emergency cesarean section		344	(66%)
Perinatal complications			
Gestational diabetes		88	(17%)
Preterm PROM		143	(28%)
Hypertensive disorder in pregnancy		330	(64%)
Fetal growth restriction		125	(24%)
HELLP syndrome		14	(3%)
Placental abruption		9	(2%)
Placental previa or vasa previa		43	(8%)
Past medical history or comorbidities			
Malignancy		10	(2%)
Prior conization or radical trachelectomy		44	(8%)
Offspring sex (female)		236	(46%)
Birth weight	(g)	1859	(257–2498)
Small for gestational age		123	(24%)
Large for gestational age		11	(2%)
Neonatal congenital anomaly		40	(8%)

BMI: Body mass index, IVF-ET: *in-vitro* fertilization-embryo transfer, PROM: Premature rupture of membrane. Data were median (range) or n (%).

### Singleton babies with LBW delivered after 37 gestational weeks

The comparison of maternal and perinatal characteristics between LBW and non-LBW (i.e., control) in the case of deliveries after 37 gestational weeks are shown in Table 4. There were no notable differences in the maternal age at delivery, incidence of nulliparity, and method of conception between the two groups. The pre-pregnancy BMI, GWG, gestational weeks at delivery, and birthweight in the LBW group were significantly lower than those in the non-LBW group. Among the perinatal complications, the prevalence of HDP and anemia during pregnancy was significantly higher in the LBW group than in the non-LBW group. The proportion of babies who were SGA was also higher in the LBW group than in the non-LBW group.

**Table 4:** Comparison of maternal and neonatal characteristics between LBW and non-LBW in mothers delivered after 37 gestational weeks.

		LBW (n = 279)		non-LBW (n = 2965)		p value
Maternal age at delivery	(years)	36	(16–56)	36	(18–62)	0.41
Maternal age at delivery≧35		182	(65)	1800	(61)	0.14
Maternal pre-pregnancy BMI	(kg/m^2^)	19.9	(14.9–33.3)	20.2	(14.7–46.5)	0.0008
Maternal pre-pregnancy BMI category						0.028
Underweight (BMI< 18.5)		68	(24)	561	(19)	
Normal weight (18.5≤ BMI< 25.0)		193	(69)	2214	(75)	
Overweight (25.0≤ BMI< 30.0)		15	(5)	150	(5)	
Obese (30≤ BMI)		3	(1)	40	(1)	
Gestational weight gain	(kg/40w)	8.9	(-3.4–21.6)	10.5	(-13.0–34.0)	<0.0001
Gestational weight gain category						<0.0001
Inadequate		210	(75)	1772	(60)	
Appropriate		56	(20)	928	(31)	
Excessive		13	(5)	265	(9)	
Smoking during pregnancy		0	(0)	0	(0)	-
Nulliparity		183	(66)	1956	(66)	0.9
Method of conception						0.23
Spontaneous conception		215	(77)	2183	(74)	
IVF-ET		64	(23)	782	(26)	
Gestational weeks at delivery	(weeks)	37	(37–41)	39	(37–41)	<0.0001
37 gestational weeks		148	(53)	577	(20)	<0.0001
Mode of delivery						<0.0001
Vaginal delivery		116	(42)	1849	(62)	
Cesarean section		163	(58)	1116	(38)	
Offspring sex (female)		167	(60)	1402	(47)	<0.0001
Perinatal complications						
Gestational diabetes		48	(17)	460	(16)	0.44
Hypertensive disorder of pregnancy		24	(9)	63	(2)	<0.0001
Anemia during pregnancy		21	(8)	124	(4)	0.017
Birth weight	(g)	2382	(1543–2498)	3040	(2500–4526)	<0.0001
Small for gestational age		109	(39)	74	(3)	<0.0001
Large for gestational age		0	(0)	422	(14)	<0.0001
Apgar score (1 minute)		8	(1–10)	8	(0–10)	<0.0001
Apgar score (5 minute)		9	(3–10)	9	(1–10)	<0.0001
Umbilical cord blood pH		7.33	(6.89–7.38)	7.31	(6.90–7.37)	0.0003
Neonatal congenital anomaly		29	(10)	101	(3)	<0.0001

BMI: Body mass index, IVF-ET: *in-vitro* fertilization-embryo transfer. Data were median (range) or n (%).

The results of the logistic regression analysis are shown in Table 5. Pre-pregnancy underweight, inadequate GWG, birth at 37 gestational weeks, CS, HDP, anemia during pregnancy, female babies, and neonatal congenital anomalies increased the risk of LBW in multiple regression models. Among the cases involving term birth, HDP had the highest odds ratio (OR) in relation to the risk of LBW in multiple regression models (OR 4.51, 95% CI 2.63, 7.72). However, IVF-ET was found to decrease the risk of LBW in this study. Of the 92 elective CS cases planned at 37 gestational weeks, elective CS was performed for 21 owing to maternal indications (i.e., low-lying placenta, maternal complications, and prior RT) and for 15 owing to fetal causes (i.e., FGR and congenital anomalies). However, the remaining 56 cases (61%) had no specific indications for the timing of delivery.

**Table 5:** Clinical risk factors of low birth weight based on univariate and multiple logistic regression analysis.

		Univariate			Multiple		
Category		Odds ratio	95%CI	p-value	Odds ratio	95%CI	p-value
Maternal age at delivery	< 20 years	1 (reference)			1 (reference)		
	over 35 year	1.21	(0.94–1.57)	0.14	1.15	(0.86–1.53)	0.34
Method of contraception	natural conception	1 (reference)			1 (reference)		
	IVF-ET	0.83	(0.62–1.11)	0.21	0.64	(0.46–0.89)	0.0075
Pre-pregnancy BMI	normal weight	1 (reference)			1 (reference)		
	underweight	1.38	(1.04–1.84)	0.028	1.42	(1.04–1.93)	0.026
Gestational weight gain	appropriate	1 (reference)			1 (reference)		
	inadequate	2.05	(1.55–2.72)	<0.0001	2.46	(1.82–3.33)	<0.0001
Birth at 37 gestational week	no	1 (reference)			1 (reference)		
	yes	4.67	(3.63–6.02)	<0.0001	3.89	(2.91–5.20)	<0.0001
Cesarean section birth	no	1 (reference)			1 (reference)		
	yes	2.33	(1.81–2.99)	<0.0001	1.42	(1.05–1.91)	0.024
Hypertensive disorder of pregnancy	no	1 (reference)			1 (reference)		
	yes	4.34	(2.66–7.06)	<0.0001	4.51	(2.63–7.72)	<0.0001
Anemia during pregnancy	no	1 (reference)			1 (reference)		
	yes	1.86	(1.15–3.01)	0.011	1.81	(1.08–3.04)	0.024
Neonatal sex	male	1 (reference)			1 (reference)		
	female	1.66	(1.29–2.13)	<0.0001	1.66	(1.28–2.17)	0.0002
Neonatal congenital anomaly	no	1 (reference)			1 (reference)		
	yes	3.29	(2.13–5.07)	<0.0001	3.24	(2.02–5.20)	<0.0001

CI: Confidence interval.

## Discussion

The present study has revealed the distribution of the causes for Japanese LBW through a single hospital-based investigation, and several causes for the birth of neonates with LBW were identified. Particularly, our results revealed that mothers with multiple pregnancies and HDP, both in the case of preterm and term birth, were at a higher risk of delivering babies with LBW.

The association between LBW and future health problems among preterm infants was analyzed in several previous articles, but these studies did not address the causes for preterm birth [1,18], and other reports regarding the causes for LBW did not even mention the gestational weeks at birth [2,3,5]. Since the effects on lifelong healthcare of children might vary depending on the causes for LBW, we were also concerned that these analyses may have included cases that should have been excluded. Furthermore, since LBW is merely classified by birthweight, a 250-g baby and a 2,499-g baby would be equally analyzed as part of the LBW group. The LBW group included babies who were just born at an earlier pregnancy, who had congenital anomalies, and who had other risk factors such as maternal complications. The report from China stated that inadequate GWG, preterm birth, pregnancy-induced hypertension, oligohydramnios, and male babies increase the risk of LBW [19]. However, the profile of LBW is still unknown in Japan. Therefore, we decided to report on the causes of LBW in babies in the hope of rekindling interest in the analysis of the prognosis of LBW infants.

In the present study, 22.5% of the LBW babies were from multiple pregnancies and 202 babies (87%) were born before 37 gestational weeks owing to various causes. The higher prevalence of LBW in multiple pregnancies than in a singleton pregnancy is owing to the higher risk of preterm birth, HDP, and preeclampsia [20]. The slope of fetal growth in twin pregnancies is lower than that in a singleton pregnancy, and LBW is known to be a complication in 50% of twin pregnancy cases [21]. Neonates born from multiple pregnancies undergo many socioeconomic, educational, and emotional problems [22,23]. Therefore, since the impact on future healthcare could differ between singleton and multiple pregnancies when the effects of LBW on healthcare are considered, it might be better to exclude multiple pregnancy cases from such analyses.

In the present study, HDP was found to be the most important cause for LBW not only in preterm birth but also in term birth. Offspring born to mothers with HDP might be at a higher risk of autism spectrum disorder and attention-deficit/hyperactivity disorder because maternal inflammation might be associated with these neonatal diseases [24]. Furthermore, HDP often coexists with FGR. Babies with FGR develop several problems, such as metabolic syndrome and renal disorders, owing to a reduced number of nephrons [25]. Among the infants who underwent FGR management and rapid postnatal catch-up growth, epigenetic changes related to metabolic syndrome were detected at several 5’-C-phosphate-G-3’ (CpG) sites [26]. When mothers developed preterm PROM, a routine prescription of antibiotics was useful for the prolongation of pregnancy and reduction of short-term neonatal morbidities [27]. However, neonates exposed to antibiotics during the second or third trimester are at a higher risk of childhood obesity [28]. In this study, CS was performed in 80% of the preterm births, and it was previously reported that CS is associated with offspring obesity [28]. Therefore, considering the future health of LBW babies, we speculated that it is important to examine why neonates are delivered with LBW.

The cause for LBW in babies born at term is the most important concern because it could potentially aid in reducing the prevalence of LBW. In this study, it was found that the proportion of babies who were SGA was 39% among singleton babies born at term. Several maternal factors, such as adolescent or advanced maternal age, pre-pregnancy maternal underweight, inadequate GWG, smoking during pregnancy, and maternal complications, have been reported to be associated with SGA [20].

Recently, the ideal body image for young women has been impacted by the widespread use of social networking sites and by the media [29]. Japan has a higher rate of underweight women than other developed countries, comprising >20% of teenagers and women in their early twenties in 2012 [30]. Previous reports have suggested that maternal underweight and inadequate GWG are the causes for SGA development in Japan [6,12,13]. Though the number of SGA in-term babies resulting from maternal underweight was low in the present study, mothers with inadequate GWG were at a higher risk of delivering in-term SGA babies. The effectiveness of informing women regarding adequate GWG before conception may improve if the risk of LBW or SGA caused by inadequate GWG is explained [31,32].

Nakashima et al. reported that the birthweight of babies conceived by frozen-thawed ET (FET) was significantly higher than those by fresh ET or by other Japanese births [33]. Since FET has recently prevailed in Japan, there might be many women opting to conceive by FET among the pregnant women with IVF-ET in this study as it was considered that IVF-ET was found to decrease the risk of LBW.

The present study suggests that the risk factors for LBW consist of both modifiable and non-modifiable factors. Some clinicians attempt to control or prevent HDP or preeclampsia using several medicines, for example, aspirin [34], metformin [35], sildenafil [36], and tadalafil [37]. However, as of 2020, there is no intervention for the complete prevention or treatment of HDP. Further, it is also not possible to prevent preterm PROM. Several fetal congenital anomalies cannot be treated *in-utero*, and neonates with fetal congenital anomalies might require medical, socioeconomic, and educational support. These factors were identified as non-modifiable factors. However, among the 92 elective CS cases planned at 37 gestational weeks in the present study, 56 cases (61%) had no specific indications for the timing of delivery. Though elective CS should be scheduled before labor onset to prevent uterine rupture, the timing of elective CS is controversial globally [38]. The National Institute for Health and Clinical Excellence guidelines recommend 39 gestational weeks as the timing for elective CS; however, this is not stated in the JSOG guidelines [15]. To prevent LBW, the timing of elective CS may need reconsideration in patients with malpresentation or histories of prior CS or uterine surgery. Maternal anemia during pregnancy was found to be a risk factor for LBW in this study, similar to previously reported findings [39,40]. Pregnant women who received prior excisional surgery for cervical lesions, including those who received RT, were at a higher risk of experiencing preterm birth [41–43]. Furthermore, several mothers had to choose to undergo late preterm delivery owing to maternal complications. Since increasing maternal age also leads to an increase in the number of mothers with various medical comorbidities and fetal congenital anomalies, preconception care might be important in preventing LBW [44–46].

There are some limitations associated with this study. First, this is a retrospective study. However, there are limited data on the distribution of the risk factors for LBW; previous reports with warnings regarding increasing numbers of babies with LBW do not include explanations as to why this number is increasing in Japan. Since our hospital is a tertiary care institution in the central area of Tokyo, Japan, several pregnant women with severe complications are referred from other clinics, thereby resulting in a higher LBW incidence than that indicated in the national data of Japan (approximately 10%). We considered that conducting the study in our hospital would be suitable for evaluating the causes for LBW and that the evidence generated in the study would add to the limited data on the causes for LBW. Second, in previous reports, women with preeclampsia had a higher risk of delivering babies with LBW than those with HDP [47,48]. However, in this study, the incidence of preeclampsia was not enough to analyze the risk of LBW. Third, the single-institution research design of this study is a limitation. Further, we could not analyze the association between LBW and maternal smoking because there were no mothers who smoked during pregnancy in this study. Therefore, we are prepared to perform further research using detailed data, including the maternal smoking status, from the JSOG database to evaluate whether our findings are generalizable to the entire Japanese population.

## Conclusions

The distribution of the background factors responsible for LBW revealed in this study suggests that LBW babies are a heterogeneous population. Strategies to reduce LBW as well as to evaluate future health risks for LBW should be considered based on the causes for LBW. An important risk factor for LBW development in both preterm and term births is HDP, a non-modifiable factor without an established effective preventive treatment. It may be difficult to achieve a rapid, ambitious reduction in LBW prevalence; however, educating women with regard to the importance of adequate pre-pregnancy body weight and GWG as well as reconsidering the timing of elective CS may be key factors that could aid in accomplishing this goal.
